# Comparison of Phylogenetic Tree Topologies for Nitrogen Associated Genes Partially Reconstruct the Evolutionary History of *Saccharomyces cerevisiae*

**DOI:** 10.3390/microorganisms8010032

**Published:** 2019-12-23

**Authors:** Manuel Villalobos-Cid, Francisco Salinas, Eduardo I. Kessi-Pérez, Matteo De Chiara, Gianni Liti, Mario Inostroza-Ponta, Claudio Martínez

**Affiliations:** 1 Departamento de Ingeniería Informática, Facultad de Ingeniería, Universidad de Santiago de Chile (USACH), Santiago 9170022, Chile; 2 Centro de Estudios en Ciencia y Tecnología de los Alimentos (CECTA), Universidad de Santiago de Chile (USACH), Santiago 9170022, Chile; 3 Millennium Institute for Integrative Biology (iBio), Santiago 7500574, Chile; 4 Instituto de Bioquímica y Microbiología, Facultad de Ciencias, Universidad Austral de Chile (UACH), Valdivia 5110566, Chile; 5 Departamento de Ciencia y Tecnología de los Alimentos, Universidad de Santiago de Chile (USACH), Santiago 9170201, Chile; 6 Université Côte d’Azur, CNRS, INSERM, IRCAN, 06107 Nice, France

**Keywords:** *Saccharomyces cerevisiae*, nitrogen associated genes, evolutionary history, tree topologies, clean lineages, representative strains

## Abstract

Massive sequencing projects executed in *Saccharomyces cerevisiae* have revealed in detail its population structure. The recent “1002 yeast genomes project” has become the most complete catalogue of yeast genetic diversity and a powerful resource to analyse the evolutionary history of genes affecting specific phenotypes. In this work, we selected 22 nitrogen associated genes and analysed the sequence information from the 1011 strains of the “1002 yeast genomes project”. We constructed a total evidence (TE) phylogenetic tree using concatenated information, which showed a 27% topology similarity with the reference (REF) tree of the “1002 yeast genomes project”. We also generated individual phylogenetic trees for each gene and compared their topologies, identifying genes with similar topologies (suggesting a shared evolutionary history). Furthermore, we pruned the constructed phylogenetic trees to compare the REF tree topology versus the TE tree and the individual genes trees, considering each phylogenetic cluster/subcluster within the population, observing genes with cluster/subcluster topologies of high similarity to the REF tree. Finally, we used the pruned versions of the phylogenetic trees to compare four strains considered as representatives of *S. cerevisiae* clean lineages, observing for 15 genes that its cluster topologies match 100% the REF tree, supporting that these strains represent main lineages of yeast population. Altogether, our results showed the potential of tree topologies comparison for exploring the evolutionary history of a specific group of genes.

## 1. Introduction

The yeast *Saccharomyces cerevisiae* (hereinafter, called “*S. cerevisiae*” or simply “yeast”) is a microorganism with wide biotechnological applications, able to conduct the alcoholic fermentation in the production of many food and beverages, particularly wine [1,2]. Moreover, *S. cerevisiae* is a workhorse for molecular biology and genetic studies, being the first eukaryotic genome to be fully sequenced [3]. Currently, the advances in sequencing technologies have allowed massive sequencing projects, making available a tremendous amount of genomic information, which covered from microorganisms to plants and human genomes [4,5,6]. In this sense, the sequencing information accumulated so far has unveil *S. cerevisiae* genome content and population structure [7], and also clarified the relationship between the different species of the *Saccharomyces* genus [8,9,10].

The first attempts to study the genetic diversity of *S. cerevisiae* were using molecular markers, such as PFGE [11,12], mitochondrial DNA digestion (RFLP-mtDNA) [13] or microsatellites [14,15]. These approaches showed a strong correlation between the molecular patterns and the geographic isolation of the yeast strains, suggesting an ecological relationship between isolates [16,17]. Similarly, the first sequencing approaches were focused on individual genes, where the sequenced information was concatenated to infer phylogeny, revealing the presence of two groups of domesticated *S. cerevisiae* strains (grape wine and sake wine strains) in comparison of isolates from nature [18].

Afterwards, the genome sequencing of 36 yeast isolates from diverse ecological origins unveiled yeast population structure and demonstrated the presence of five clean lineages (defined in terms of presenting unique private SNPs not shared between lineages) within the species: Wine/European (WE), West African (WA), North American (NA), Sake (SA) and Malaysian (MA) [19]. Subsequent massive sequencing projects have confirmed these results, including the 100 yeast genomes project [20] and the 1002 yeast genomes project [21]. This last project represents the most complete catalogue of yeast genetic diversity so far, expanding the number of phylogenetic clusters initially observed in the species to 26 clades [19,21].

After the initial insights into yeast population structure [19], five strains were considered as representatives of the first five clean lineages identified: DBVPG6765 (WE strain) for WE cluster, DBVPG6044 (WA strain) for WA cluster, YPS128 (NA strain) for NA cluster, Y12 (SA strain) for SA cluster and UWOPS03.461.4 (MA strain) for MA cluster. Between them, the MA strain has been seldom utilised in genetic studies, due to its reproductive isolation respect to the other four representative strains [22,23]. Thus, this set of four strains has become a powerful tool for disentangling the genetic basis of quantitative traits in yeasts, allowing to map the causative alleles of phenotypic variation [23,24]. In this context, the WE, WA, NA and SA strains have been utilised as founder (parental) strains of recombinant yeast populations, which have permitted the mapping of QTLs (Quantitative Trait Loci) for multiple phenotypes [25,26,27,28,29,30].

Given that fermentative phenotypes are in general quantitative traits, different strains have been used to map QTLs related to the fermentation process, such as: fermentation rate [31], aroma compounds production [32] and nitrogen consumption [33,34,35]. Among these phenotypes, our group has systematically focused its interest on nitrogen associated phenotypes, due to the importance of nitrogen sources for the fermentation process, being its deficiencies the principal cause of stuck and sluggish fermentations (reviewed in [36,37]). In this sense, we have mapped multiple QTLs related to nitrogen consumption using yeast populations derived from the four representative strains (WE, WA, NA and SA), validating the specific causative genes by reciprocal hemizygosity approaches [35,38,39,40]. However, it is not fully understood which fraction of the genetic diversity observed in the species is represented by the alleles mapped in these QTL experiments, making necessary performing bioinformatic analyses to assess these contributions.

One bioinformatic approach is phylogenetic inference, which attempts to reconstruct a hypothesis that explains the evolutionary relationships between a group of species, strains, genes or proteins. The historical pattern of speciation and divergence allows classifying life according to an evolutionary schema which usually is represented as a phylogenetic tree [41]. For instance, the reconstruction of the tree of life represents the evolutionary relationship between millions of species [42]. Phylogenetic inference has been used in almost every branch of biology, such as botany [43], zoology [44], palaeontology [45] and pharmacology [46], among other fields [47]. Furthermore, phylogeny has been applied to describe the relationships between paralogue gene families, understanding the evolution and epidemiological dynamics of pathogens, studying cell differentiation in cancer and other diseases, identification of gene function, prediction of the protein tertiary structure, metagenomic sequences classification, and reconstruction of ancestral genomes [48].

Despite the advances in molecular phylogenetic reconstruction methods, the inference process always involves some uncertainty respect to the true historical relationships of the organisms, and phylogeny may include incongruences (i.e., conflicting topologies of the trees for a same taxa) related to analytical or biological factors [41,49]. The analytical factors include biases associated to (i) the selection of the inference criterion, (ii) taxon sampling, and (iii) specific assumptions in the modelling of sequence evolution. Biological factors are related to (i) the biological evidence used to infer phylogeny, (ii) the presence of reticular evolution phenomena (e.g., horizontal gene transfer, incomplete lineage sorting, gene duplication, hybridisation, recombination, among others), (iii) stochastic errors or character sampling biases related to the length of the genes, and (iv) systematic errors due to the presence of noise in the dataset [50]. One way of addressing the biases produced by the mentioned factors is dealing with the problem using a multi objective optimisation approach [51]. However, a complete phylogenetic study requires a comparative analysis to reduce the uncertainty associated to the inference process, understanding the sources of incongruence, and assessing their effects on the resultant tree topologies [50].

In this work, we evaluated the potential of phylogenetic tree topologies comparison for evolutionary history reconstruction of a given set of genes in *S. cerevisiae*. For a proof of concept, we selected 22 genes related to nitrogen consumption phenotypes under fermentation conditions, all of them previously mapped by linkage analyses. For this set of genes, we analysed the sequence information from 1011 yeast strains that were part of the “1002 yeast genomes project”. Utilising the concatenated sequences of these genes, we partially reconstructed the evolutionary history of the species, obtaining a phylogenetic tree with 27% of topology similarity respect to the reference tree (REF) described by [21] (using whole genome sequencing). Additionally, we generated individual phylogenetic trees for each selected gene and compared the tree topologies among them, observing genes with similar tree topologies and suggesting a similar evolutionary history between them. Finally, we pruned the constructed phylogenetic trees to compare topologies between each phylogenetic cluster/subcluster within the population or between a specific subset of strains. Altogether, our results are a proof of concept that shows how tree topologies comparison can be used to explore the global evolutionary history of the species, opening the possibility of a wider evaluation, encompassing the entire *S. cerevisiae* genome.

## 2. Materials and Methods

### 2.1. Gene Selection and Genomic Information Obtention

We selected 22 genes involved in nitrogen associated phenotypes during the fermentation process, identified by QTL mapping and validated by reciprocal hemizygosity analyses (i.e., with a demonstrated effect over the phenotype) [26,35,38,39,40,52]. Importantly, most of the selected genes were mapped using yeast populations where the founder strains are the DBVPG6765 (WE), DBVPG6044 (WA), YPS128 (NA) and Y12 (SA) strains [22,23]. The list of genes selected, including their functional information, QTL detected and bibliographic reference, is shown in Supplementary Table S1.

The genomic information for the 22 selected genes was obtained from the “1002 yeast genome project” [21]. A VCF (Variant Calling Format) file containing the genotype information of the 22 selected genes was transformed into linear sequences utilising a custom script (please see next section). The DBVPG6765 (WE) strain was not included in the “1002 yeast genomes project” and its sequencing information for the 22 selected genes was extracted from SGRP2 Blast server (http://www.moseslab.csb.utoronto.ca/sgrp/) [53].

### 2.2. Bioinformatic Analyses

Computational experiments were performed using R version 3.3.2 and RStudio version 0.99.491 in an Intel (R) Core (TM) i7-3930K CPU 3.20 GHz, 6 cores, 16 GB RAM and 2TB. Computational experiments consider three stages: (i) data processing, (ii) phylogenetic inference, and (iii) comparison analysis.

#### 2.2.1. Data Processing and Phylogenetic Inference

A custom script based on the *vcfR* package v1.8.0 [54] was used to process the 22 gene sequence variations for 1011 strains stored as a VCF file. For each gene, its sequence was compared to the reference genome and produced a multiple sequence alignment using the information of the 1011 strains, which was stored as an individual FASTA file. Then, an integrated phylogenetic tree (Total Evidence tree, TE) was inferred using the direct concatenation of the sequences for these 22 genes stored as different files, according to the total evidence paradigm [55]. This paradigm, also called “combined analysis” or “congruence approach”, combines all the data sets associated to diverse biological evidence before the inference of a phylogenetic tree, for example, by concatenating sequences of multiple genes [56]. This is considered a good alternative when the data sets are statistically congruent (i.e., similar evolutionary rates or non-conflicting phylogenetic trees) [49].

The TE tree was constructed using the Neighbor-joining strategy, following the same procedure described by [21], by applying the *bionj* algorithm available in the *phangorn* R package v2.5.5 [57]. This strategy was used to infer 22 phylogenetic trees which represent the individual evolutionary history of each gene. In addition, the congruence of these individual trees was calculated by using the Congruence Among Distance Matrix (CADM) metric [49]. This metric computes a coefficient of concordance W (0≤W≤1), where 0 represents a complete disagreement between the input trees and 1 means complete agreement.

#### 2.2.2. Comparison of the Phylogenetic Tree Topologies

A quantitative comparison between phylogenetic trees was performed. To do this, a similarity matrix of trees was built by calculating the percentage of clades in common (PC) [58], $PC=100\times \left[1-\frac{RF}{\left(2m-6)\right)}\right]$, where the RF is the topological Robinson-Foulds distance between trees and *m* is the number of strains. The RF distance corresponds to the minimum number of editions (merging or splitting nodes) necessary to transform one unrooted tree into a second tree [59]. Then, the Multidimensional Scaling method, available in the *treespace* R package v1.1.3.1 [60], was used to graphically compare the tree topologies by exploring the landscape of phylogenetic trees. Two types of comparisons were performed; first, the topologies of all the trees produced were compared: the TE tree, the REF described by [21], the individual trees related to the 22 selected genes, and a randomly created topology (OUT). The OUT tree was inferred to be contrasted with the REF tree, in order to assure that the differences (or similarities) between trees are produced by the feature of the data instead of noise or bias associated to the inference methods.

Then, all trees generated (TE, REF, OUT and individual trees for each gene) were pruned using the *phangorn* R package to perform specific comparisons between (i) the subtrees (cluster/subcluster) associated to the clades defined by [21] and (ii) the representative strains of the clean lineages WE, WA, NA and SA [19]. The RF metric compares trees inferred using the same set of elements (strains), thus, the tree topologies coming from the DBVPG6765 (WE) strain were not compared respect to the REF tree, since this strain was not included by [21].

## 3. Results and Discussion

### 3.1. Reconstructing the Population Structure of *S. cerevisiae* Using a Subset of Nitrogen Associated Genes

Initially, we selected 22 genes associated with the fermentation capacity (fermentation kinetic measured as $CO_{2}$ release) and nitrogen consumption (ammonium or amino acids) phenotypes under wine fermentation conditions. These genes were mapped using linkage analysis approaches (QTL mapping) and all of them were validated by reciprocal hemizygosity analyses, confirming their effects over the analysed phenotypes (Supplementary Table S1). Furthermore, the 22 selected genes were identified utilising yeast populations derived from four strains (WE, NA, WA and SA strains) representative of previously described yeast clean lineages [19,23]. Therefore, it is expected that the selected genes represent alleles coming from the main phylogenetic clusters observed in the *S. cerevisiae* population, encompassing a great fraction of the genetic diversity of the species [19].

**Figure 1:**
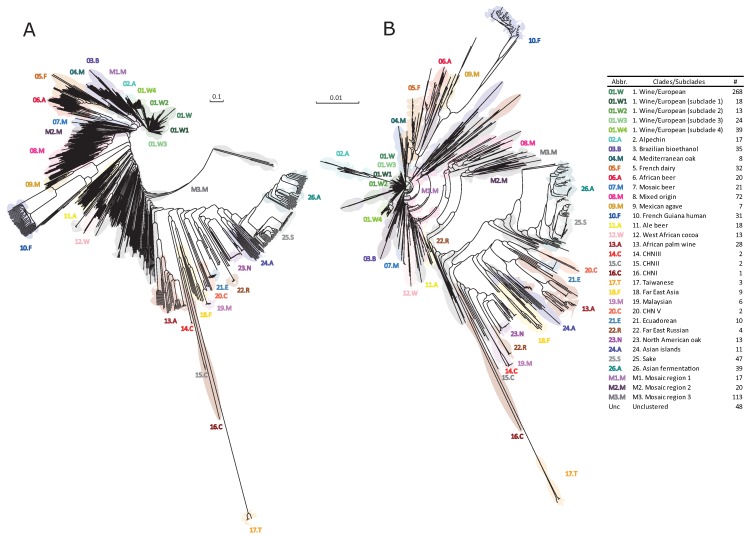
Population structure of *S. cerevisiae* obtained with total and partial genomic information. (**A**) Neighbor-joining tree described by [21] using the whole genome sequencing information of 1011 yeast strains. This tree was considered as the Reference (REF) tree. (**B**) Neighbor-joining tree obtained with the concatenated information of the 22 selected genes in 1011 yeast strains. This tree was considered as the Total Evidence (TE) tree. In both trees the phylogenetic clusters and subclusters are represented by the same colour code.

**Figure 2:**
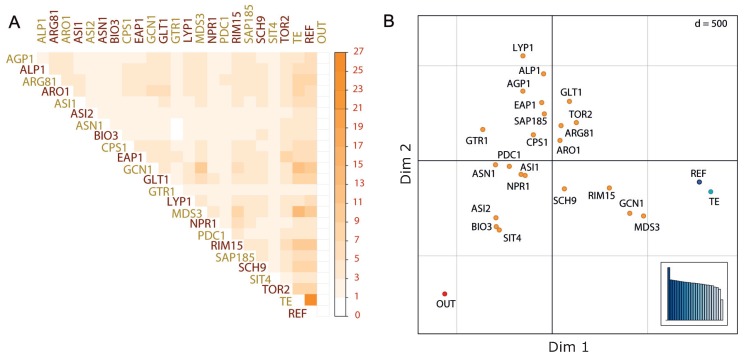
Comparison of tree topologies for nitrogen associated genes. (**A**) Similarity matrix comparing tree topologies between genes. The REF and TE trees were included as controls, in addition to the randomly generated OUT tree. The colour-scale goes between the minimum (0%, white) and maximum (27%, dark orange) similarities observed between individual trees. (**B**) Bi-dimensional representation of the tree spaces obtained from the topology comparison. The multidimensional scaling was performed by using the *Smacof* R package [62].

In order to assess how much of the evolutionary history of the species can be reconstructed with this subset of 22 genes, we used the genomic information from the “1002 yeast genome project” [21]. This allowed us to analyse the genetic information for the 22 selected genes in 1011 different yeast strains isolated from diverse ecological origins. We concatenated the sequence information of these 22 genes and performed a phylogenetic inference to construct a TE tree (Figure 1A). The CADM metric was 0.6, meaning that the evolutionary histories of the genes studied are congruent, supporting the use of the total evidence paradigm in the phylogenetic analysis according to the definition used by [49]. Then, we compared the structure of the TE tree respect to the REF tree described by [21] using whole genome sequencing (Figure 1B), observing in both trees a similar topology with the same set of clades and subclades (Figure 1). We quantified the topology differences observed between the TE and REF trees using the topological Robinson-Foulds distance, showing a 27% of similarity between trees (Figure 2A), an interesting new result with no bibliographic antecedent which marks a comparison value for future studies of the same type. Altogether, these results showed that partial genomic information from nitrogen associated genes partially reconstructed (27%) the evolutionary history of the species. This result clearly contrasts with the obtained for the OUT tree, a randomly created topology that showed no similarity with TE and REF trees (Figure 2A).

### 3.2. Comparison of Tree Topologies among Genes Revealed Similar Evolutionary Histories

Afterwards, we generated individual trees for each selected gene using the information from the 1011 strains, and then we compared the tree topologies by means of Robinson-Foulds distance among them using as controls the TE and REF trees, in addition to an output (OUT) tree randomly generated (Figure 2A). In general, we observed groups of genes with similar tree topologies, highlighting the tree topologies of *GCN1*, *MDS3* and *RIM15*, which also are the closer ones to the TE and REF trees (Figure 2B). One possible explanation is that these genes have the higher contribution to the topologies of the TE and REF trees, which would imply a stronger contribution in the population structure of the species (Figure 2). However, is important to note that the length and/or conservation degree of the genes under study may introduce some bias. Since we used only the polymorphic sites present in each gene (and not the total length of each one), a potential bias may come only from the conservation degree (understood as the number of polymorphic sites), and this could be evaluated only for the 22 genes used in this proof of concept. A more wide and detailed study, e.g., using all *S. cerevisiae* genes, is necessary to gain a better understanding at this respect.

Other strong association is observed between *EAP1* and *TOR2*, both genes participating of the TORC1 signalling pathway (Figure 2). Interestingly, molecular diversity among the WE, NA, WA and SA strains for TORC1 activation have been recently revealed [61]. However, other TORC1 pathway associated genes (*GTR1*, *NPR1*, *SAP185*, *SCH9* and *SIT4*) showed more different tree topologies, with a disperse localisation in the tree space plot representation in 2D (Figure 2B). Overall, our results confirmed that comparison of tree topologies among different genes is a useful tool to identify genes with similar evolutionary histories.

We further analysed our data set (22 genes in 1011 strains) comparing the tree topologies within each cluster and subcluster described by [21] (REF tree). For this, we pruned all the trees constructed (individual gene, TE and REF trees), allowing us to compare topologies between clades with the same elements (tips or strains). After the pruning, we compared the topology of each cluster and subcluster observed in the REF tree respect to the topology of the cluster and subcluster present in individual gene trees and TE tree (Supplementary Table S2). For some clusters, we observed genes with an apparently strong contribution on its evolutionary history (Supplementary Table S2). This is because, while all genes have their own contribution to the evolutionary history of the species, some of them have a discordant contribution. For instance, for the African beer cluster (that includes 20 strains), the *LYP1* gene reconstructs 59% of the cluster topology (cluster evolutionary history) in the REF tree, meaning that the topology of the African beer cluster for *LYP1* gene is 59% identical to the African beer cluster observed in the REF tree (Supplementary Table S2). Interestingly, for the Wine/European cluster (that includes 268 strains), the TE tree including information for the 22 genes studied reconstructs 25% of the cluster topology in the REF tree (Supplementary Table S2; see subclade 0 and TE column). This result suggests that our topology comparison may be biased by the number of strains present in each cluster/subcluster (Supplementary Table S2), where a low number of strains within a cluster/subcluster increases the probability of inferring the same tree topology. For example, while using four strains it is possible to reconstruct 3 unrooted and 15 rooted trees, increasing the number of strains to six increases the number of possible topologies to 105 unrooted and 945 rooted trees. This bias was reduced by applying the PC metric which includes the normalised RF scores (see Section 2.2.2). Altogether, our results spotted genes with a possible strong contribution to the cluster/subcluster topology observed in the REF tree. Importantly, the OUT tree (Figure 2 and Supplementary Table S2), included as a control, results different to all the other tree without clades/subclades in common.

### 3.3. Representative Strains from Clean Lineages Reconstruct the Evolutionary History of the Species

Finally, we compared tree topologies in a subset of strains which have been considered as representative of clean lineages within the species (WE, WA, SA and NA) since the first insights into yeast population structure [19]. With now a larger number of strains that have been sequenced and the identification of a total of 26 clades [21], the question of whether these four strains still fully encompass the sequence variation observed in these genes across the entire *S. cerevisiae* population arises. We used again the pruned versions of the constructed trees (individual gene, TE and REF trees) to compare topologies between trees that contain the same elements, but in this case, we compared the topology of the REF tree respect to the topology present in the individual gene trees and TE tree considering only four representative strains of these clean lineages (Supplementary Table S3).

For this, we initially selected four strains generally used as representatives of these *S. cerevisiae* clean lineages: DBVPG6765 (WE), DBVPG6044 (WA), YPS128 (NA) and Y12 (SA) [19,23]. However, since topology comparison among trees requires the same elements, the DBVPG6765 strain was replaced in the TE tree by the DBVPG1106 strain, a genetically similar strain also belonging to the Wine/European cluster. This was due to the absence of the DBVP6765 strain in the “1002 yeast genome project” (Supplementary Figure S1). Then, we proceed with the topology comparison among trees only considering the information from these strains (Table 1 and Supplementary Table S3). We observed for the TE tree and 15 individual gene trees that their topologies were 100% identical to the REF tree (Table 1 and Supplementary Table S3), suggesting that information for these four representative strains reflects the global evolutionary history of the species. We performed as a control the same analysis using the information for the 22 genes of six strains isolated from Chile by our lab group that belong to Wine/European cluster (subclade 0; Supplementary Table S2), and that were included in the “1002 yeasts genome project”. The results showed for all the genes evaluated a 0% of identity respect the REF tree (Table 1 and Supplementary Table S3), suggesting that these strains are not able to reconstruct the evolutionary history observed in the REF tree, which is an expected result since these strains belongs to the same clade and are probably very similar genetically. Altogether, our results confirmed the ability of these four representative strains (DBVPG1106, DBVPG6044, K12 and YPS128) to reconstruct the global evolutionary history of the species, an astounding idea considering that the number of clusters have expanded up to 26 [21]. Nevertheless, other complementary approaches (e.g., analysis of non-synonymous SNPs across the “1002 yeast genomes project” strains) could help to better support this idea.

**Figure 3:**
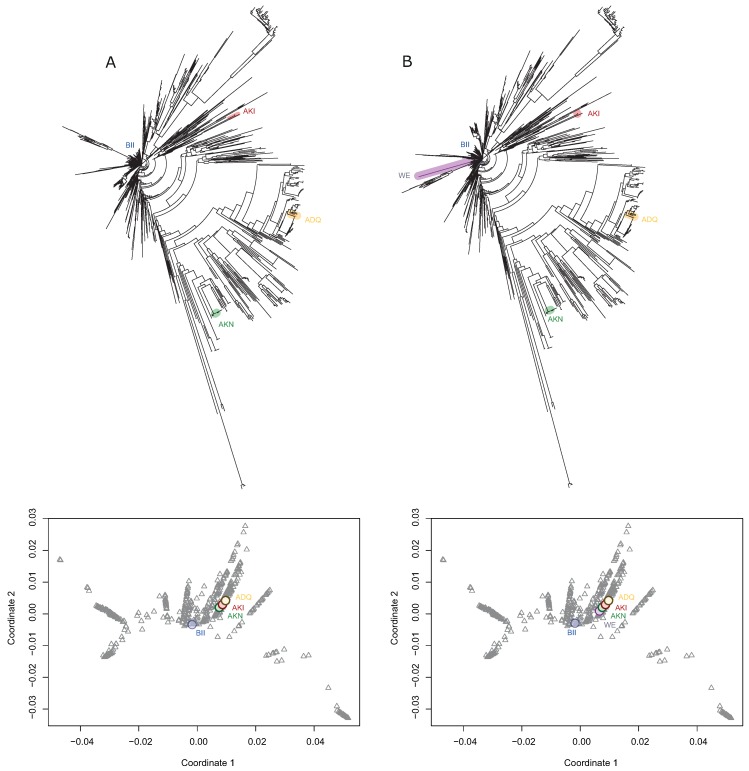
Representative yeast strains in the context of the population structure of the species. (**A**) TE tree highlighting the position of four strains (BII: DBVPG1106; AKI: DBVPG6044; ADQ: K12 and AKN: YPS128), which are representative of four yeast clean lineages. (**B**) TE tree considering also the DBVPG6765 (WE) strain. Below each TE tree bi-dimensional tree space representation is showed, obtained from the phylogenetic distance matrix among strains. The positions of the representative strains are highlighted.

Since we replaced in tree topologies comparison the DBVPG6765 strain by the DBVPG1106 strain as representative of the Wine/European cluster, we included the DBVPG6765 strain in the TE tree using the information from the SGRP database (Figure 3A). The DBVPG6765 strain is grouped with the Wine/European clade but seems to have a greater phylogenetic distance: this is probably due to the inclusion of the *RIM15* gene within the analysis, since the DBVPG6765 strain carries a unique allele with polymorphisms not present in other strains of its clade [26,53]. Afterwards, we localised the representative strains in the 2D tree space plot, observing that DBVPG6765, DBVPG6044, K12 and YPS128 strains were grouped together, being separated from the DBVPG1106 strain and distant to other strains (Figure 3B and Supplementary Figure S1). This suggests that DBVPG6765, DBVPG6044, K12 and YPS128 strains have a similar phylogenetic distance between them (Figure 3B), supporting its use as representative strains of clean lineages in *S. cerevisiae*, which in turn validate all previous efforts using this set of four strains to perform QTL mapping studies. Interestingly, this conclusion echoes some previous results obtained using a similar approach but at a smaller scale [63].

In conclusion, the comparison of tree topologies for nitrogen associated genes allowed us to partially reconstruct the evolutionary history of the species and identify genes with similar evolutionary trajectories. We also compared the topologies for each phylogenetic cluster or subcluster present in the REF tree, observing genes with cluster/subcluster topologies of high similarity to the REF tree. Finally, we compared tree topologies for a subset of representative strains from clean lineages present in the *S. cerevisiae* population structure, showing that topologies for the TE tree and 15 individual gene trees match in 100% the REF tree topology, supporting the idea that these representative strains reflect the global population structure of the species. Overall, we have shown the potential to assess the evolutionary history of the species by combining tree topologies comparison and the “1002 yeast genomes project” information, which in turn may lead to a more thorough exploration of *S. cerevisiae* evolution at a genomic level. Analysis of genes from other functional groups would help deepen in the usefulness of tree topology comparison to infer similar evolutionary histories for genes associated with a particular cellular function and, in this context, this proof of concept opens the possibility to perform similar analysis using the whole *S. cerevisiae* genome, and then examine the evolutionary patterns of multiple genes with different cellular functions, confirming or rejecting that genes with similar functions have similar evolutionary histories.

## Figures and Tables

**Table 1:** Tree topologies comparison considering representative strains (DBVPG1106, DBVPG6044, K12 and YPS128) and Chilean strains. N: Number of strains. TE: Total Evidence phylogenetic tree. REF: Reference phylogenetic tree. OUT: Randomly generated tree.

Strain/Origin	N	TE	REF	OUT
Others	1001	27	100	0
Chile	6	0	100	0
Representatives of clean lineages	4	100	100	0

## Supplementary Materials

The following are available online at https://www.mdpi.com/2076-2607/8/1/32/s1.
