# Supplementary material for: Comparison of Phylogenetic Tree Topologies for Nitrogen Associated Genes Partially Reconstruct the Evolutionary History of Saccharomyces cerevisiae
Source: Microorganisms. 2019 Dec 23;8(1):32. doi: 10.3390/microorganisms8010032 (PMC7022669; doi:10.3390/microorganisms8010032)
Supplement: Supplementary file 1 [file microorganisms-08-00032-s001.zip › Definitions/logo-mdpi-eps-converted-to.pdf]

MDPI
