# Supplementary material for: Comparison of Phylogenetic Tree Topologies for Nitrogen Associated Genes Partially Reconstruct the Evolutionary History of Saccharomyces cerevisiae
Source: Microorganisms. 2019 Dec 23;8(1):32. doi: 10.3390/microorganisms8010032 (PMC7022669; doi:10.3390/microorganisms8010032)
Supplement: Supplementary file 1 [file microorganisms-08-00032-s001.zip › microorganisms-SI.pdf]

microorganisms

MDPI

Article

Comparison of Phylogenetic Tree Topologies for Nitrogen Associated Genes Partially Reconstruct the Evolutionary History of *Saccharomyces cerevisiae*

Manuel Villalobos-Cid [1], Francisco Salinas [2,3,4], Eduardo I. Kessi-Pérez [2,5], Matteo De Chiara [6], Gianni Liti [6], Mario Inostroza-Ponta [1] and Claudio Martínez [2,5,*]

* Correspondence: claudio.martinez@usach.cl

Received: 14 November 2019; Accepted: 19 December 2019; Published: 23 December 2019

Appendix S1. Supplementary material

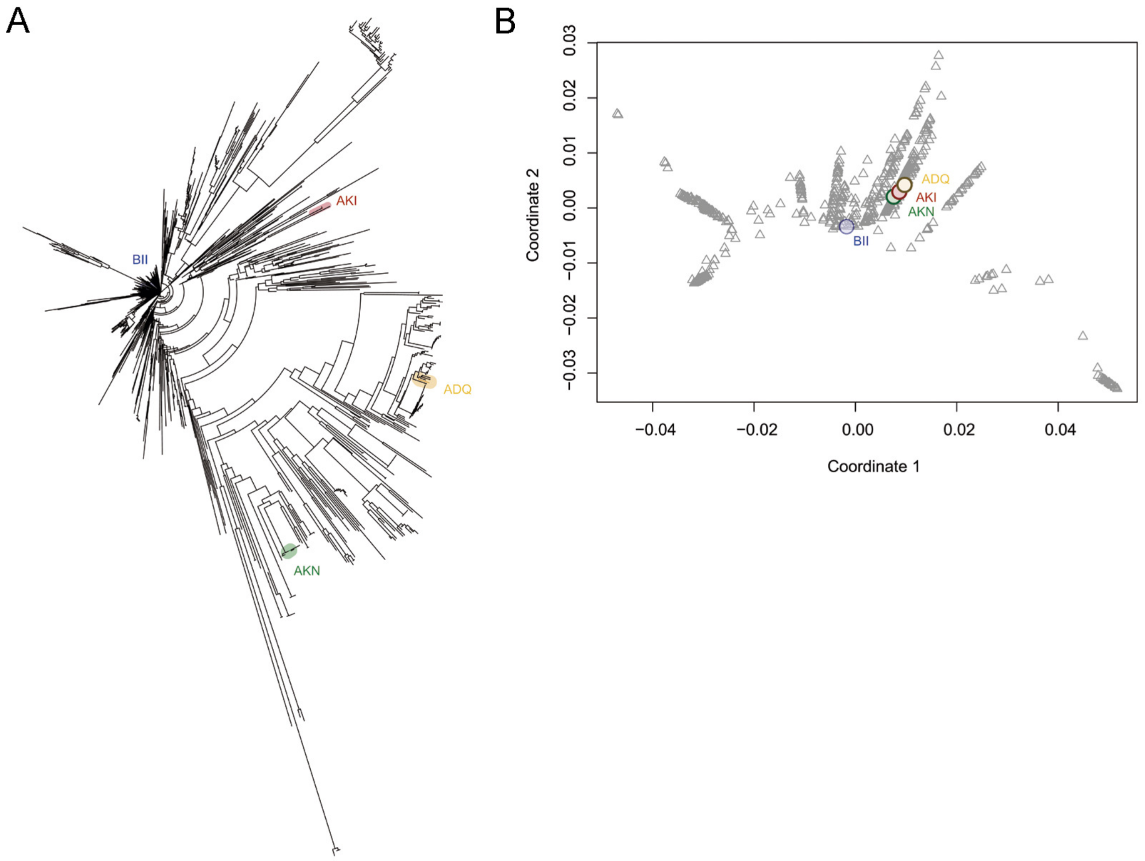

Figure S1. Representative yeast strains in the context of the population structure of the species. (a) TE tree highlighting the position of four strains (BII: DBVPG1106; AKI: DBVPG6044; ADQ: K12 and AKN: YPS128), which are representative of four yeast clean lineages. (b) TE tree represented as bi-dimensional tree spaces, obtained from the phylogenetic distance matrix among strains. The positions of the representative strains are highlighted.

Table S1. Genes evaluated in this work. ASE: Allele specific expression; ASB: Allele-specific transcription factor binding; BSA: Bulked segregant analysis; ISA: Individual segregant analysis.

ARG81	Zinc finger transcription factor involved in arginine-responsive genes	Fermentative capacity	BSA	[1]
BIO3	7,8-diamino-pelargonic acid aminotransferase (DAPA)	Fermentative capacity	BSA	[1]
GCN1	Positive regulator of the Gcn2p kinase activity	Fermentative capacity	BSA	[1]
MDS3	Putative component of the TOR regulatory pathway	Fermentative capacity	BSA	[1]
AGP1	Low-affinity amino acid permease with broad substrate range	Nitrogen consumption	ISA	[2]
ASI1	Subunit of the inner nuclear membrane Asi ubiquitin ligase complex	Nitrogen consumption	ISA	[2]
GLT1	NAD(+)-dependent glutamate synthase (GOGAT)	Nitrogen consumption	ISA	[2]
RIM15	Protein kinase involved in cell proliferation in response to nutrients	Fermentative capacity	ISA	[3]
ASN1	Asparagine synthetase	Nitrogen consumption	ASE, ASB	[4]
ARO1	Pentafunctional arom protein	Nitrogen consumption	ISA	[5]
ALP1	Arginine transporter	Nitrogen consumption	ISA	[5]
ASI2	Subunit of the inner nuclear membrane Asi ubiquitin ligase complex	Nitrogen consumption	ISA	[5]
CPS1	Vacuolar carboxypeptidase S	Nitrogen consumption	ISA	[5]
LYP1	Lysine permease	Nitrogen consumption	ISA	[5]
PDC1	Major of three pyruvate decarboxylase isozymes	Nitrogen consumption	ISA	[5]
EAP1	eIF4E-associated protein, competes with eIF4G for binding to eIF4E	Nitrogen consumption	ISA	[6]
GTR1	Subunit of a TORC1-stimulating GTPase and the EGO/GSE complex	Nitrogen consumption	ISA	[6]
NPR1	Protein kinase, stabilizes several plasma membrane amino acid transporters	Nitrogen consumption	ISA	[6]
SAP185	Protein that forms a complex with the Sit4p protein phosphatase	Nitrogen consumption	ISA	[6]
SCH9	AGC family protein kinase, phosphorylated by Tor1p	Nitrogen consumption	ISA	[6]
SIT4	Ceramide-activated, type 2A-related serine-threonine phosphatase	Nitrogen consumption	ISA	[6]
TOR2	PIK-related protein kinase, subunit of TORC1 and TORC2	Nitrogen consumption	ISA	[6]

Table S2. Tree topologies comparison for the subclades previously described by [7]. N: Number of strains. TE: Total Evidence phylogenetic tree. REF: Reference phylogenetic tree. OUT: Randomly generated tree.

Clades/Subclades	N	*AGP1*	*ALP1*	*ARG81*	*ARO1*	*ASI1*	*ASI2*	*ASN1*	*BIO3*	*CPS1*	*EAP1*	*GCN1*	*GLT1*	*GTR1*	*LYP1*	*MDS3*	*NPR1*	*PDC1*	*RIM15*	*SAP185*	*SCH9*	*SIT4*	*TOR2*	TE	REF	OUT
1. Wine/European (subclade 0)	268	1	4	7	6	5	2	1	3	4	5	9	4	1	2	8	3	3	9	4	6	3	7	25	100	0
1. Wine/European (subclade 1)	18	0	13	7	13	13	0	0	0	0	7	13	0	0	0	0	0	0	20	7	7	0	20	20	100	0
1. Wine/European (subclade 2)	13	0	0	0	0	0	0	0	0	0	0	0	0	0	0	0	0	0	0	0	0	0	0	9	100	0
1. Wine/European (subclade 3)	24	0	0	0	0	0	0	0	0	0	0	0	0	0	0	0	0	0	0	0	0	0	0	0	100	0
1. Wine/European (subclade 4)	39	5	0	8	8	0	0	0	0	0	0	8	2	0	0	2	0	0	8	0	0	0	0	36	100	0
2. Alpechin	17	2	16	2	9	9	9	0	2	0	9	45	37	2	30	23	2	0	52	9	9	2	30	73	100	0
3. Brazilian bioethanol	35	6	3	3	6	0	0	0	3	9	12	3	3	0	3	0	0	12	3	0	9	0	9	12	100	0
4. Mediterranean oak	8	0	0	0	0	0	0	0	0	0	0	0	0	0	0	0	0	0	0	0	0	0	0	0	100	0
5. French dairy	32	0	7	3	3	7	7	0	10	10	10	3	7	0	7	3	0	7	10	7	7	3	14	17	100	0
6. African beer	20	12	47	29	35	12	12	6	24	12	24	24	41	6	59	24	29	24	24	35	53	12	24	59	100	0
7. Mosaic beer	21	6	11	11	0	17	11	6	0	0	11	0	11	22	11	11	11	6	17	6	22	6	11	44	100	6
8. Mixed origin	72	0	1	1	0	0	1	0	0	0	1	1	0	1	4	1	0	3	0	3	0	0	0	1	100	0
9. Mexican agave	7	0	0	20	0	0	0	0	0	0	0	0	0	0	0	0	0	0	20	0	0	20	0	0	100	0
10. French Guiana human	31	4	0	11	4	4	4	0	4	0	0	0	4	0	0	4	4	0	4	4	0	4	0	14	100	0
11. Ale beer	18	0	13	13	13	40	20	13	33	7	20	33	13	13	13	20	27	13	7	0	7	7	7	40	100	0
12. West African cocoa	13	7	0	27	0	7	0	0	0	0	0	7	7	7	0	7	0	7	7	7	0	7	0	27	100	0
13. African palm wine	28	16	20	20	20	12	8	16	28	20	16	24	36	16	20	16	12	28	24	24	28	4	36	40	100	0
14. CHNIII	2	-	-	-	-	-	-	-	-	-	-	-	-	-	-	-	-	-	-	-	-	-	-	-	-	-
15. CHNII	2	-	-	-	-	-	-	-	-	-	-	-	-	-	-	-	-	-	-	-	-	-	-	-	-	-
16. CHNI	1	-	-	-	-	-	-	-	-	-	-	-	-	-	-	-	-	-	-	-	-	-	-	-	-	-
17. Taiwanese	3	-	-	-	-	-	-	-	-	-	-	-	-	-	-	-	-	-	-	-	-	-	-	-	-	-
18. Far East Asia	9	0	21	0	0	0	5	0	0	0	0	5	0	5	0	0	0	0	21	0	5	0	5	38	100	0
19. Malaysian	6	0	0	0	0	0	0	15	0	0	0	0	0	0	0	0	0	0	0	0	0	0	0	0	100	0
20. CHN V	2	-	-	-	-	-	-	-	-	-	-	-	-	-	-	-	-	-	-	-	-	-	-	-	-	-
21. Ecuadorean	10	0	0	0	0	0	0	0	0	0	0	0	0	0	0	0	0	0	0	0	0	0	0	0	100	0
22. Far East Russian	4	100	100	100	100	100	100	100	100	100	100	100	100	100	100	100	100	100	100	100	100	100	100	100	100	0
23. North American oak	13	0	0	0	0	0	0	0	0	0	0	0	0	0	0	0	0	0	0	0	0	0	0	0	100	0
24. Asian islands	11	2	2	2	2	27	2	2	14	2	0	0	14	0	0	27	0	0	14	2	0	0	2	64	100	0
25. Sake	47	2	5	0	11	2	0	0	0	0	7	5	9	7	0	9	2	0	14	2	2	0	11	20	100	0
26. Asian fermentation	39	19	6	8	8	11	8	19	3	3	8	19	11	3	8	19	3	3	11	6	14	6	19	33	100	0
M1. Mosaic region 1	17	21	14	0	21	14	0	14	0	7	36	14	14	7	7	14	14	14	29	14	14	14	14	21	100	0
M2. Mosaic region 2	20	12	18	29	12	18	29	18	12	12	6	18	24	0	18	12	18	18	18	18	18	6	6	24	100	0
M3. Mosaic region 3	113	7	9	14	10	7	4	5	5	5	10	13	11	2	8	10	9	4	15	7	12	3	14	26	100	0
Unclustered	48	20	22	27	24	22	13	16	11	16	16	29	22	9	27	27	13	9	29	20	24	13	18	44	100	0
Total strains	1011																									

Table S3. Tree topologies comparison for the strains representative of the clean lineages previously described by [8]. N: Number of strains. TE: Total Evidence phylogenetic tree. REF: Reference phylogenetic tree. OUT: Randomly generated tree.

Strain/Origin	N	*AGP1*	*ALP1*	*ARG81*	*ARO1*	*ASI1*	*ASI2*	*ASN1*	*BIO3*	*CPS1*	*EAP1*	*GCN1*	*GLT1*	*GTR1*	*LYP1*	*MDS3*	*NPR1*	*PDC1*	*RIM15*	*SAP185*	*SCH9*	*SIT4*	*TOR2*	TE	REF	OUT
Unclustered	1001	5	6	8	6	5	4	3	4	5	6	9	8	2	5	10	4	4	10	6	7	3	8	27	100	0
Chile	6	0	0	0	0	0	0	0	0	0	0	0	0	0	0	0	0	0	0	0	0	0	0	0	100	0
Representatives of clean lineages	4	100	100	100	0	100	0	0	0	100	100	100	100	100	100	100	100	100	0	100	100	0	0	100	100	0

N: Number of strains. TE: Total Evidence phylogenetic tree. REF: Reference phylogenetic tree. OUT: Randomly generated tree.

References

1. Brice, C.; Sanchez, I.; Bigey, F.; Legras, J.L.; Blondin, B. A genetic approach of wine yeast fermentation capacity in nitrogen-starvation reveals the key role of nitrogen signaling. *BMC Genom.* **2014**, *15*, 495.
2. Jara, M.; Cubillos, F.A.; García, V.; Salinas, F.; Aguilera, O.; Liti, G.; Martínez, C. Mapping Genetic Variants Underlying Differences in the Central Nitrogen Metabolism in Fermenter Yeasts. *PLoS ONE* **2014**, *9*, e86533.
3. Kessi-Pérez, E.I.; Araos, S.; García, V.; Salinas, F.; Abarca, V.; Larrondo, L.F.; Martinez, C.; Cubillos, F.A. RIM15 antagonistic pleiotropy is responsible for differences in fermentation and stress response kinetics in budding yeast. *FEMS Yeast Res.* **2016**, *16*, fow021.
4. Salinas, F.; de Boer, C.G.; Abarca, V.; Garcia, V.; Cuevas, M.; Araos, S.; Larrondo, L.F.; Martinez, C.; Cubillos, F.A. Natural variation in non-coding regions underlying phenotypic diversity in budding yeast. *Sci. Rep.* **2016**, *6*, 21849.
5. Cubillos, F.A.; Brice, C.; Molinet, J.; Tisné, S.; Abarca, V.; Tapia, S.M.; Oporto, C.; García, V.; Liti, G.; Martínez, C. Identification of Nitrogen Consumption Genetic Variants in Yeast Through QTL Mapping and Bulk Segregant RNA-Seq Analyses. *G3 Genes Genomes Genet.* **2017**, *7*, 1693–1705.
6. Molinet, J.; Cubillos, F.A.; Salinas, F.; Liti, G.; Martinez, C. Genetic variants of TORC1 signaling pathway affect nitrogen consumption in Saccharomyces cerevisiae during alcoholic fermentation. *PLoS ONE* **2019**, *14*, e0220515.
7. Peter, J.; De Chiara, M.; Friedrich, A.; Yue, J.X.; Pflieger, D.; Bergstrom, A.; Sigwalt, A.; Barre, B.; Freel, K.; Llored, A.; et al. Genome evolution across 1,011 Saccharomyces cerevisiae isolates. *Nature* **2018**, *556*, 339–344.
8. Liti, G.; Carter, D.M.; Moses, A.M.; Warringer, J.; Parts, L.; James, S.A.; Davey, R.P.; Roberts, I.N.; Burt, A.; Koufopanou, V.; et al. Population genomics of domestic and wild yeasts. *Nature* **2009**, *458*, 337–341.

© 2019 by the authors. Licensee MDPI, Basel, Switzerland. This article is an open access article distributed under the terms and conditions of the Creative Commons Attribution (CC BY) license (http://creativecommons.org/licenses/by/4.0/).
